# 

**Published:** 1892-07

**Authors:** 


### CONTENTS.

ORIGINAL COMMUNICATIONS—

The Surgical Treatment of Puerperal Peritonitis. By W. E. B. Davis, M. D., Rome, Ga. .......... 257

Atrophic Rhinitis. By Charles D. Roy, A. B., M. D., Atlanta, Ga. .......... 262

What is Gynecology? By R. R. Kime, M. D., Atlanta, Ga. .......... 270

The Treatment of Abortion and Some of the Complications Incident Thereto. By Walter A. Crow, M. D., Atlanta, Ga .......... 276

SOCIETY REPORTS—

Clinical Society of Maryland .......... 278

Allegheny County Medical Society .......... 282

CORRESPONDENCE—

New York Letter .......... 285

EDITORIAL—

The American Medical Association .......... 290

Death of Dr. L. P. Kennedy .......... 296

Money Value of Medical Attendance .......... 298

SELECTIONS .......... 299

MEDICAL ITEMS .......... 315

BOOK REVIEWS .......... 318

INDEX TO ADVERTISEMENTS .......... xii

ITEMS OF INTEREST .......... xi

